# Impact of the COVID-19 Pandemic on a Physician Group’s WhatsApp Chat: Qualitative Content Analysis

**DOI:** 10.2196/31791

**Published:** 2021-12-07

**Authors:** Sawsan Abdel-Razig, Pascale Anglade, Halah Ibrahim

**Affiliations:** 1 Cleveland Clinic Abu Dhabi Abu Dhabi United Arab Emirates; 2 Cleveland Clinic Lerner College of Medicine Case Western Reserve University Clevelend, OH United States; 3 Department of Medicine Sheikh Khalifa Medical City Abu Dhabi United Arab Emirates; 4 Johns Hopkins University Baltimore, MD United States

**Keywords:** WhatsApp, social media, physician, pandemic, COVID-19, qualitative, communication, misinformation, information-seeking behavior, information seeking, information sharing, content analysis, community

## Abstract

**Background:**

Social media has emerged as an effective means of information sharing and community building among health professionals. The utility of these platforms is likely heightened during times of health system crises and global uncertainty. Studies have demonstrated that physicians’ social media platforms serve to bridge the gap of information between on-the-ground experiences of health care workers and emerging knowledge.

**Objective:**

The primary aim of this study was to characterize the use of a physician WhatsApp (WhatsApp LLC) group chat during the early months of the COVID-19 pandemic.

**Methods:**

Through the lens of the social network theory, we performed a qualitative content analysis of the posts of a women physician WhatsApp group located in the United Arab Emirates between February 1, 2020, and May 31, 2020, that is, during the initial surge of COVID-19 cases.

**Results:**

There were 6101 posts during the study period, which reflected a 2.6-fold increase in platform use when compared with platform use in the year prior. A total of 8 themes and 9 subthemes were described. The top 3 uses of the platform were requests for information (posts: 2818/6101, 46.2%), member support and promotion (posts: 988/6101, 16.2%), and information sharing (posts: 896/6101, 14.7%). A substantial proportion of posts were related to COVID-19 (2653/6101, 43.5%), with the most popular theme being requests for logistical (nonmedical) information. Among posts containing COVID-19–related medical information, it was notable that two-thirds (571/868, 65.8%) of these posts were from public mass media or unverified sources.

**Conclusions:**

Health crises can potentiate the use of social media platforms among physicians. This reflects physicians’ tendency to turn to these platforms for information sharing and community building purposes. However, important questions remain regarding the accuracy and credibility of the information shared. Our findings suggest that the training of physicians in social media practices and information dissemination may be needed.

## Introduction

### Background

The COVID-19 pandemic has put an unprecedented and prolonged strain on health systems and health care providers globally. Clinicians are inundated with global developments, an incessant news cycle, and minute-by-minute information from various sources, as new and sometimes conflicting data are becoming available worldwide at an unparalleled pace [1]. The evolving and shifting nature of public health policies, including curfews, lockdowns, social distancing restrictions, and testing and tracing requirements, presents additional challenges. In order to ensure their personal safety and provide care, frontline medical professionals need to be equipped with the most evidence-based clinical pathways and public health protocols.

### Use of Social Media

Several studies have shown that physicians’ social media platforms have become significant facilitators of bridging gaps of information between on-the-ground experiences of health care workers and emerging, scientific, clinical, and population-level knowledge [2,3]. Researchers have analyzed various social media platforms to better understand their use in public health discourse [4,5]. Moreover, infoveillance studies have confirmed a marked increase in individuals’ activity on social media platforms, particularly during the COVID-19 pandemic [6]. For example, a COVID-19 physician group, which was created on Facebook in March 2020 and was described as “an inclusive resource for physicians to share front line clinical information about COVID-19 as it becomes available,” quickly rose to considerable popularity; the group has approximately 29,000 members to date [7]. Other studies have described how health care providers worldwide have used Twitter and WhatsApp (WhatsApp LLC) during the pandemic to disseminate news and discoveries to colleagues and communicate health information directly to patients [2,8]. There is limited published information however on the use of social media among groups of physicians during a medical and public health crisis.

We previously reported an analysis of the WhatsApp posts of a women physician group; we noted that the platform was effective in enabling female physicians to expand networks, exchange ideas, share scientific information, celebrate accomplishments, and provide support to colleagues [9]. In this study, by using social network theory as an overarching lens [10], we sought to analyze the content of the social media interactions of this group’s members during a public health crisis. The primary purpose of this study was to characterize the use of a social media platform among members of a physician group during the COVID-19 pandemic. Our additional aims included examining group members’ levels of engagement (ie, by comparing them to members’ prepandemic levels of engagement) and identifying the sources of medical information that were shared among the physician members.

## Methods

### Setting and Population

On January 23, 2020, the United Arab Emirates (UAE) reported its first confirmed case of COVID-19. Over the following 4 months, the country experienced a surge of cases; over 60,000 UAE patients were infected with SARS-CoV-2.

WONDER (Women Doctors in the Emirates) is a multispecialty women physician group that originated in 2015 to foster support and collaboration among female physicians living and working in the UAE. In early 2018, WhatsApp Messenger (WhatsApp LLC)—a closed-group messaging app—was added as an adjunct to face-to-face meetings. At the time of writing this paper, group membership totaled 161 physicians, and over 80% (130/161, 80.7%) of members wrote posts during the study period.

### Data Collection and Analysis

Posts from February 1, 2020, through May 31, 2020, were included in the analysis. February 1, 2020, corresponds to the national index case of COVID-19, and the studied period encompasses the initial spring 2020 COVID-19 surge in the UAE, during which the number of COVID-19 cases and related hospitalizations peaked [11]. Prior to data extraction and analysis, group members were informed about this retrospective study via a WhatsApp message and were given the opportunity to have their posts excluded from analysis. Data were exported from the WhatsApp Messenger group to Microsoft Office Excel 2013 by one of the researchers (PA), who removed identifying information; retained the content, dates, and times of posts; and assigned each member a unique numeric identifier to calculate the percentage of members who wrote posts. All data were then anonymized for qualitative analysis. The messages were analyzed via qualitative content analysis [12]. We approached the data through the lens of the social network theory, which focuses on the effect of social relationships on processing media influence, transferring information, and enabling attitudinal or behavioral change [10]. Two physician researchers (HI and PA) independently coded each post, performed a content analysis of the messages, and produced a list of the common themes that they identified. After this initial review, the researchers discussed their findings, and through discussion, they reached consensus on the themes and created subthemes. The two primary reviewers then independently categorized all posts according to the predetermined themes. Any disagreements were resolved by consensus, and any remaining discordance was brought to the third physician researcher (SAR) and discussed until consensus was achieved. A descriptive quantitative analysis was conducted by using Microsoft Excel 2019 to analyze the frequency of posts within each identified theme. This study was reviewed by the Cleveland Clinic Abu Dhabi Research Ethics Committee and was deemed exempt from institutional review board review, as the data were retrospective, were deidentified, and did not involve any patient information.

### Team Reflexivity

We were cognizant that our research team consisted of 3 female physicians who lived and worked in the Middle East and were frontline workers during the COVID-19 pandemic. To minimize bias, we were blinded to participants’ identities. We were mindful of how our experiences influenced our analysis of the data and engaged in frequent group conversations to share, support, and challenge each other’s interpretations.

## Results

At the time of data extraction and analysis, there were 161 group members—a 32% increase from the 122 members in the prior year. From February 1, 2020, to May 31, 2020, there were a total of 6101 posts. Of the 161 members, 130 (80.7%) posted at least once during this time period. The number of posts increased 2.6-fold from the number of posts during the same time period in 2019. Further, 1204 more messages were posted in the chat during this 5-month study interval than during the entire preceding year (6101 posts vs 4897 posts, respectively). Approximately half of all posts (2653/6101, 43.5%) were directly related to the COVID-19 pandemic.

There were 8 general themes identified. Table 1 provides a description of themes and subthemes, example posts, and the relative frequencies of each theme. The most frequent theme was related to requests for information, which represented 46.2% (2818/6101) of all posts. Of the information requests, the majority (1308/2818, 46.4%) were requests for non–COVID-19–related general medical information, and many of these posts consisted of physician referral requests. Of the COVID-19–related information requests, the vast majority (945/1184, 79.8%) were related to logistical information, including quarantine measures, school closures, or mask mandates. Only 3.9% (239/6101) of all posts consisted of specific diagnostic or treatment queries regarding COVID-19.

Approximately 15% (896/6101, 14.7%) of the posts consisted of medical information that was shared with the group by individual members. There were twice as many posts containing information from unverified and non–evidence-based sources (n=517; eg, blogs, social media messages, and local newspaper articles) as there were posts containing evidence-based information (n=297). Several group members expressed confusion and frustration. One group participant noted the following:

I have a headache from all the COVID-19 stuff I am reading. I no longer know what to trust and who to believe.

Another physician stated:

This article a perfect example about why a lot of the social media posting of drafts, small series, unproven theories, personal opinion etc. is frankly dangerous. We should defer to only published peer reviewed papers and guidelines. I am personally overwhelmed with all the misinformation I get.

The frequencies of posts related to each COVID-19 subtheme are displayed in Table 2. In total, 35.6% (945/2653) of COVID-19–related posts were requests for logistical information, and 22.7% (601/2653) of such posts contained supportive or promotional messages related to COVID-19.

**Table 1:** Themes derived from the qualitative analysis of the WhatsApp group chat (posts: N=6101).

Social network theory principles, general themes, and subthemes					Description of theme			Posts, n (%^a^)			Example posts
**Relationships in the context of general information seeking**											
	**Request for information**			Member requesting information from other group members			2818 (46.2)			N/A^b^	
		General (medical)	Information sought on any non–COVID-19–related medical matter			1308 (21.4)			“I need a recommendation for a fertility/ IVF center in Abu Dhabi.” “Any pediatric urologist in the group, or one that can be recommended?”		
		COVID-19 logistics	Information sought on logistical topics, such as the location of testing sites, curfew rules, and personal protective equipment protocols			945 (15.5)			“Question for the OB in the group: What are your PPE protocol in labor and delivery, patient of unknown COVID status? Any guidelines or protocols on that?” “Does this mean no school for 4 weeks starting this Sunday?”		
		General (nonmedical)	Information sought on any nonmedical matter			326 (5.3)			“Dear ladies, any houseplant experts in the group, particularly orchids.” “Any recommendations for piano repair who will visit your home?”		
		COVID-19 medical	Information sought on COVID-19 diagnoses, symptoms, and treatment protocols			239 (3.9)			“Good evening ladies Is there any evidence that fasting and or associated dehydration is a risk favor for worse outcomes in covid19? Just wondering as Ramadan is almost upon us.”		
**Relationships in the context of community building**											
	**Support and promotion**			Member providing moral or emotional support to other group members			988 (16.2)			N/A	
		COVID-19 related	Support for COVID-19–related matters			601 (9.9)			“Just wanted to thank you all for your support and prayers, my uncle was extubated yesterday, he’s officially off the vent but we know he’s not out of the woods yet. Hopefully no long-term sequelae from this infection and he’ll be able to go back to his family soon.”		
		General	Support given for any non–COVID-19–related matters			387 (6.3)			“Watch interview with our very own member educating the public on the crucial role anesthesiologists play in delivering safe patient care.”		
	Community engagement			Non–medical-related general posts, quotes, memes, videos, or articles of interest			637 (10.4)			“I second your gratitude for all the blessings we have...and most importantly each other. Once this is over, I plan to make hugs mandatory amongst all Wonder members”	
	Celebration			Secular and religious holiday wishes, personal and professional milestones, and celebrations			350 (5.7)			“Beautiful baby! Wishing him a life full of happiness, health and prosperity.”	
	Group administration			Posts for announcing group activities and events as well as for adding and welcoming new group members			250 (4.1)			“Reminder: Our Zoom meet is tonight at 730p. We have a dozen WONDER docs signed up already. Anyone else wants to join? DM me your email address. Looking forward to catching up!”	
	Women empowerment			Articles regarding women in medicine, inspirational quotes, and images related to women's empowerment			73 (1.2)			“Meet the top 10 Power Businesswomen in the Middle East' ranked by Forbes”	
**Relationships in the context of information sharing**											
	**Information sharing**			Member sharing information with the group			896 (14.7)			N/A	
		COVID-19 non–evidence-based source	COVID-19–related information from social media sources			571 (9.4)			“…this is from a Romanian physician Facebook group. Seems credible…”		
		COVID-19 evidence-based source	COVID-19–related information from peer-reviewed literature and verifiable sources			297 (4.9)			“A great summary interview with Bruce Aylward of the WHO.” “Published today in NEJM: in hospitalized adult patients with severe Covid-19, no benefit was observed with lopinavir–ritonavir.”		
		General medical	General medical information from any source			28 (0.5)			“You are cordially invited to attend Allergy Connect with Experts.“		
	Opportunities			Member seeking or offering employment, volunteer, or donation opportunities			89 (1.5)			“Abu Dhabi Blood Bank is running low on reserves as so few people have gone in to donate over the past month. If you know donors, please encourage them to go.” “I have a friend who’s a gynecologist in London and looking for a position in Dubai or Abu Dhabi. Please let me know if anyone knows of any vacancies.”	

^a^Percentages are rounded to the nearest tenth. Cumulative percentages may not equal 100%.

^b^N/A: not applicable.

**Table 2:** Analysis of the subset of posts related to COVID-19 (n=2653).

Subtheme	COVID-19–related posts, n (%)
Requests for logistical information related to COVID-19	945 (35.6)
Requests for medical information related to COVID-19	239 (9)
Sharing of COVID-19–related information from peer-reviewed literature and verifiable sources	297 (11.2)
Sharing of COVID-19–related information from social media and unverifiable sources	571 (21.5)
Supportive, encouraging, or promotional messages related to COVID-19	601 (22.7)

## Discussion

### Use of Social Media

Social media content can provide important insights into the issues and topics that concern health care providers during a global health crisis. Our report on the use of a physician group’s WhatsApp chat during the evolving COVID-19 pandemic demonstrates the increased use of this social media forum, and a substantial proportion of the content was related to COVID-19. Social network theory emphasizes the importance of relationships in the context of general information seeking, knowledge sharing, and community building [10]. Accordingly, our study identified 3 main aims of the use of the WhatsApp group, namely information requests, information dissemination, and support and encouragement. A major premise of the social network theory is that a wide network of weaker relationships allows for access to more individuals and resources and can therefore be more beneficial than 1 or 2 strong ties [10]. Throughout the early months of 2020, health care workers cared for large volumes of critically ill patients without any evidence-based therapies while simultaneously dealing with an onslaught of research findings and information [1]. Our findings reflect the confusion and frustration that are often felt by frontline physicians who are trying to navigate a global public health emergency for themselves, their families, and their patients. In our study, group chat members often turned to their colleagues for advice and support. This was evidenced by the large volume of conversations that occurred during this time period and the high engagement levels of members. Compared to the number of posts from the same time period in the previous year, the number of posts increased substantially in 2020. Although there was a modest increase in membership, the exponential rise in the number of posts likely represents the increased use of the platform as a resource for physicians during the extraordinary circumstance of a global pandemic. In fact, 1204 more posts were found during our 5-month study interval than during the entire preceding year (6101 posts vs 4897 posts, respectively). Other studies have reported increased social media use during the COVID-19 pandemic [2,3,6,9].

Almost half (2653/6101, 43.5%) of the posts were directly related to COVID-19, and over one-third (945/2653, 35.6%) of these posts consisted of requests for logistical information. The non–COVID-19–related posts highlighted several important points. For instance, even in the midst of a global pandemic, physicians continued to provide general medical care to their patient populations. Additionally, group members frequently discussed non–COVID-19–related and nonmedical interests. As such, the chat group likely allowed for the opportunity to provide a sense of normalcy to frontline workers in the midst of a public health crisis. It is interesting that the vast majority of information requests related to COVID-19 dealt with logistics rather than with medical or treatment queries (945/1184, 79.8%). This may have been due to the lack of information available at the time about the novel SARS-CoV-2, but this may also signify the general confusion on public health protocols, which grew as lockdowns, school closures, and social distancing restrictions were implemented. In addition to personally navigating these regulations, physicians were required to guide patients through testing, isolation, and quarantine procedures, often with limited medical knowledge and under frequently changing government policies. Further, as the studied posts were from a women physician group, it should be noted that the group members likely had primary caregiver roles within their families and were personally impacted by the COVID-19 public health protocols. Studies have confirmed that women physicians often bear the majority of childcare and household responsibilities [13,14]. The focus of posts on COVID-19–related logistics may therefore reflect day-to-day priorities that may be shared among female group members. It is notable that community engagement, celebration, and promotion collectively remained important themes during the pandemic. This reflects the tendency of group members to encourage and support each other during uncertain times, which further reinforces the critical role of social media platforms in facilitating a sense of community among medical professionals.

### Study Implications

We are concerned that a substantial majority (571/868, 65.8%) of the posts in the WhatsApp group that contained COVID-19–related information often cited media, social media, or unnamed sources. In fact, only one-third (297/868, 34.2%) of such posts contained information from published medical literature or other verifiable sources. The sharing and discussion of various medical messages within a physician group could serve as a means of critiquing the veracity of information or creating awareness of web-based misinformation. However, the sheer volume of unverified posts and the frequent deviations from the evidence-based data that physicians are expected to disseminate could result in confusion and the inadvertent spread of misinformation from physicians to their patients, as evidenced by several chat participants who expressed concern and frustration about the posting of unverified information by other group members. This phenomenon is not unique to this one chat group. A previous study on the prevalence of misinformation in tweets about health care found that approximately 20% of tweets were inaccurate [15]. In fact, one of the most widely spread conspiracy theories, which linked COVID-19 to the 5G network, was traced to comments made by a Belgian physician in January 2020 [16]. The director-general of the World Health Organization described the large volume of unproven or inaccurate information on social media during the pandemic as an *infodemic of misinformation* [17]. The confusion resulting from misinformation can ultimately cause physicians to question the legitimacy of new scientific discoveries regarding effective COVID-19 therapeutics or vaccines. Moreover, the high volume of social media posts can result in information fatigue. Despite the understandable desire of physicians to share and receive useful information, without verifying the veracity and credibility of information prior to sharing it, physicians risk unwittingly facilitating the dissemination of misinformation. This may reflect a lack of formal social media education among physicians [18], which is compounded by the considerable challenges of analyzing data during an evolving pandemic [19]. More and more social interactions are occurring on web-based platforms. As such, physicians should be more cognizant of appropriate and effective social media use in the context of data analysis, synthesis, and sharing. Our findings have several important implications. First, the results substantiate the critical role that social media platforms play in facilitating communication and fostering connectedness among physicians coping with population health crises. Second, our study also provides insights on content and topics that seem to be the most relevant to physician communities during such crises. Lastly, our data reflect areas of concern regarding the use of social media in these professional communities during times of uncertainty and can be used to inform the design of future interventions and research.

In addition to confirming the results of prior work, this study highlights the need for additional research into the evidence-based approaches that physicians use to analyze health information obtained from social media. Similar concerns have been raised about the increasing dissemination of medical information through social media, including the lack of editorial oversight for web-based data and the harm caused by the rapid dissemination of incorrect medical information [20].

### Limitations

Although this study involves a single international physician group, the findings likely reflect the common challenges faced by health care workers who deliver health services in dynamic logistical and biomedical environments that are intensified by global health crises. The study group contained only women physicians; therefore, our findings may represent priorities that correlate with gendered experiences and roles. To our knowledge, the existing literature lacks published studies that report on gender differences in physicians’ social media behaviors, though studies have suggested that there are gender differences in success on social media platforms [21,22]. Lastly, it is unknown whether group messages actually impacted physicians’ attitudes or behaviors.

### Conclusion

The uncertainties posed by an evolving global health crisis represent considerable challenges to the health care workforce. As the world has become increasingly more connected through social media, these platforms represent critical information dissemination tools. Our findings confirmed the importance of social media in creating a communicative and collaborative platform for physicians in the midst of a public health emergency. Although more accessible information can undoubtedly benefit patient care, our findings raised important questions regarding the accuracy and credibility of shared information. Larger multinational infoveillance studies are needed to better understand social media discourse among physicians during public health crises.

## Abbreviations

UAE: United Arab Emirates
WONDER: Women Doctors in the Emirates
